# Absence of Scleroderma pattern at nail fold capillaroscopy valuable in the exclusion of Scleroderma in unselected patients with Raynaud’s Phenomenon

**DOI:** 10.1186/s12891-016-1206-5

**Published:** 2016-08-15

**Authors:** Lesley-Anne Bissell, Giuseppina Abignano, Paul Emery, Francesco Del Galdo, Maya H. Buch

**Affiliations:** 1 Leeds Institute of Rheumatic and Musculoskeletal Medicine, University of Leeds, 2nd Floor, Chapel Allerton Hospital, Chapeltown Road, Leeds, LS7 4SA UK; 2 NIHR Leeds Musculoskeletal Biomedical Research Unit, Leeds Teaching Hospitals NHS Trust, Leeds, UK

**Keywords:** Systemic sclerosis, Nail-fold capillaroscopy, Raynaud’s Phenomenon

## Abstract

**Background:**

To report the predictive value of nail-fold capillaroscopy (NFC) patterns of vasculopathy for systemic sclerosis (Scleroderma; SSc) in an unselected cohort of patients with Raynaud’s phenomenon (RP).

**Methods:**

Patients referred to a tertiary SSc clinic with RP were evaluated by light/video-NFC. Clinical diagnosis, details and serology were recorded. Primary RP was defined as RP with no features of connective tissue disease (CTD)/antibody. NFC patterns were determined: normal, non-specific, ‘early’, ‘active’ or ‘late’ SSc patterns. Fulfilment of the VEDOSS or 2013 ACR/EULAR criteria for SSc was determined following NFC assessment.

**Results:**

Three hundred forty-seven patients were referred: mean (SD) age 47 (15.2) years. On clinical review, 54 (16 %) did not have RP, 69 (20 %) had primary RP, 52 (15 %) had SSc and 172 (50 %) had secondary RP. NFC SSc pattern was detected in 80 (23 %) patients; 37/52 with SSc, 30/172 with secondary RP, 9/69 with primary RP and 4/54 with no RP. For identifying patients who met either the VEDOSS or 2013 ACR/EULAR criteria for SSc, detection of a SSc NFC pattern had a sensitivity of 71 %, specificity 95 %, positive predictive value 84 % and negative predictive value 90 %.

**Conclusions:**

The absence of SSc NFC pattern in patients with RP or suspected CTD is very valuable in the exclusion of SSc.

## Background

Nail-fold capillaroscopy (NFC) is a non-invasive method of evaluating the microcirculation in vivo, developed and utilised to characterise disease since the early 20^th^ century [1, 2]. Primary RP (pRP) is associated with normal architecture whereas abnormalities are recognised in association with secondary RP. The most well described abnormalities in NFC have been in patients with systemic sclerosis (SSc), with the recognition of the SSc NFC pattern; presence of early, active or late vasculopathy [3]. The SSc NFC pattern correlates with SSc disease duration [3], severity [4], and is predictive of future organ damage [5].

NFC has been shown to be helpful in the assessment of pRP and predicting progression to connective tissue disease (CTD) [6–8]; a meta-analysis reported any abnormal capillaroscopy pattern offered a positive predictive value of 47 % and negative predictive value of 93 % for developing CTD [9]. In patients with pRP, a SSc NFC pattern, in particular, was reported by Pavlov-Dolijanovic et al, to offer a negative predictive value of 99 % for the future development of SSc, with an odd ratio of 163 (95 % confidence interval: 97.9, 271.5) [10].

However, although recognised to occur, few studies have reported the prevalence of a SSc NFC pattern in the context of an unselected cohort with secondary RP (sRP) [11–14]. The largest study of 447 patients reported a SSc NFC pattern in 65 % of 102 SSc patients, 13 % of 186 patients with RP (primary and secondary combined together), 14 % with undifferentiated CTD (UCTD), 27 % with dermato-/poly-myositis and 8.5 % with Systemic Lupus Erythematosus (SLE) [13]. Due to this lack of data, the predictive value of a SSc NFC pattern for the development of SSc in patients presenting with sRP is unknown.

In 2013, the American College of Rheumatology (ACR)/European League Against Rheumatism (EULAR) classification criteria for SSc were developed in an effort to overcome the relatively low sensitivity of the existing SSc criteria, particularly in early disease and limited cutaneous SSc [15]. Assigning varying weights to specific features of disease, including fingertip lesions, telangiectasia, abnormal NFC and SSc-related autoantibodies, sensitivity and specificity was improved to 91 % and 92 % respectively. In addition, the VEDOSS (Very Early Diagnosis of Systemic Sclerosis) diagnostic criteria for early SSc were produced, with an aim to detect those at the early stages of the disease not yet meeting the ACR/EULAR criteria [16]. In these criteria, the presence of RP along with anti-nuclear antibody (ANA) and SSc NFC pattern are major criteria. Therefore, given that a SSc NFC pattern contributes to meeting both these new criteria the potential lack of specificity for SSc warrants further exploration.

Our objective was to describe, in real-life clinical practice, the association of a SSc NFC pattern with clinical diagnosis in an unselected cohort of patients with RP attending a tertiary centre NFC service. We also wished to determine the predictive value of a SSc NFC pattern for meeting either the 2013 ACR/EULAR or VEDOSS criteria for SSc.

## Methods

Patients with RP are referred to our specialist SSc tertiary centre for NFC to aid in the diagnosis, prognosis and management of RP. Patients with RP that were referred for NFC between January 2009 and October 2013 formed the basis for this study. The cohort is unselected and includes both pRP and sRP.

At the time of NFC, clinical diagnosis, RP history and serological status were used to define patients were defined as having: (i) pRP - RP with no features of CTD/antibody positivity (ii) sRP - a clinical diagnosis of non-SSc CTD, such as systemic lupus erythematosus (SLE) or undifferentiated CTD (UCTD); but also included patients with features suspicious of (but not fulfilling formal diagnosis of) SSc [such as the presence of anti-nuclear antibody (ANA) or another ‘red flag’ feature, namely gastro-eosophageal reflux disease (GORD), digital ulceration, telangiectasia, or puffy fingers)] (iii) SSc (clinical diagnosis).

Patients were acclimatised to room temperature for at least 10 min before NFC examination. A small drop of immersion oil was placed on the nail beds of the eight fingers (excluding thumbs) and the nail-fold capillaries were examined by either light microscopy or with the availability of newer technology, video-microscopy. The examination was carried out by a physician with a specialist interest in SSc. NFC patterns were determined as: normal, non-specific, or ‘early’, ‘active’ or ‘late’ SSc pattern as described by Cutolo previously [3].

Findings were systematically recorded. A retrospective analysis of the clinical records determined the fulfilment of the VEDOSS criteria for early SSc or 2013 ACR/EULAR criteria for SSc for all patients following NFC assessment.

A descriptive analysis was carried out using SPSS (version 21, IBM, NY, USA) statistical package.

## Results

### Patient characteristics

Three hundred forty-seven patients were referred to the NFC service between January 2009 and October 2013; mean (SD) age was 47 (15.2) years, 83 % were female. Table 1 describes the patient characteristics. On clinical review, 54 (16 %) were determined not to have true RP, 69 (20 %) had pRP, 172 (50 %) had sRP, and 52 (15 %) had a clinical diagnosis of SSc. Of the patients with SSc, 29 (56 %) were anti-centromere antibody (ACA) positive, 4 (8 %) anti-topoisomerase antibody (Scl-70) positive; 35 (67 %) had limited cutaneous SSc (lcSSc), 9 (10 %) dcSSc, 6 (11.5 %) undefined and 6 (11.5 %) overlap; at referral, 46 (89 %) patients met either VEDOSS criteria or 2013 ACR/EULAR criteria for SSc. Of those with sRP, 71 (41 %) were being managed for CTD or inflammatory arthritis whilst 101 (59 %) had either presence of an antibody and/or a red-flag feature for SSc.

**Table 1:** Patient characteristics

Variable	No RP	Primary RP	Secondary RP [subset with antibody or red flag feature]	SSc	All patients
Number, *n* (%)	54 (15.6)	69 (19.9)	172 (49.6) [101 (29.1)]	52 (15.0)	347 (100)
Age, years, mean (SD)	42.2 (14.6)	40.6 (13.9)	47.4 (14.0) [45.3 (14.8)]	58.8 (14.4)	47 (15.2)
Female	47 (87)	51 (74)	145 (84) [86 (85)]	45 (87)	288 (83)
Duration of RP, months, median (IQR)	-	61.0 (24.0, 149.0) (*n* = 64)	57 (18.0, 160.0) [60 (24.0, 180.0)] (*n* = 154 [94])	70.0 (30.0, 240.0) (*n* = 47)	60.0 (24.0, 166.0) (*n* = 265)
Current smokers, *n* (%)	9/41 (22)	17/57 (30)	40/146 (27) [22/87 (25)]	7/42 (17)	73/286(26)
Ab positive, *n* (%)	33 (61)	0 (0)	145 (84) [91 (90)]	48 (92)	226 (65)
ANA positive, *n* (%)	32 (59)	0 (0)	142 (83) [88 (87)]	48 (92)	222 (64)
ACA positive, *n* (%)	4 (7)	0 (0)	20 (12) [12 (12)]	29 (56)	53 (15)
Scl-70 positive, *n* (%)	3 (6)	0 (0)	7 (4) [3 (3)]	4 (8)	14 (4)

*Ab* antibody, *ACA* anti-centromere antibody, *ANA* antinuclear antibody, *IQR *inter-quartile range, *N* number, *RP *Raynaud’s Phenomenon, *Scl-70* anti-topoisomerase antibody, *SD* standard deviation, *SSc* Systemic Sclerosis

### NFC findings

A SSc NFC pattern was detected in 80 (23 %) patients: 43 with ‘early’, 31 with ‘active’ and six with ‘late’ pattern of vasculopathy (see Table 2). A SSc NFC pattern was seen in 37 (71 %) patients with SSc, 30 (17 %) with sRP, 9 (13 %) with pRP and 4 (7 %) with no RP. Considering only those with non-SSc CTD or inflammatory arthritis; 16 (23 %) of the 71 patients had a SSc NFC pattern; two of five patients with systemic lupus erythematosis, 8 of 42 patients with undifferentiated CTD, four of six patients with mixed CTD, one of three patients with Sjogren’s syndrome and 1 of 14 patients with inflammatory arthritis.

**Table 2:** NFC patterns seen across diagnoses

		No RP (*n* = 54)	Primary RP (*n* = 69)	Secondary RP (*n* = 172) [subset with red flag features (*n* = 101)]	SSc (*n* = 52)	All patients (*n* = 347)
NFC findings	Normal	29 (54)	27 (39)	80 (47) [51 (51)]	7 (14)	143 (41)
	Non-specific	21 (39)	33 (48)	62 (36) [36 (36)]	8 (15)	124 (36)
	Early vasculopathy	4 (7)	9 (13)	17 (10) [10 (10)]	13 (25)	43 (12)
	Active vasculopathy	0 (0)	0 (0)	11 (6) [3 (3)]	20 (39)	31 (9)
	Late vasculopathy	0 (0)	0 (0)	2 (1) [1 (1)]	4 (8)	6 (2)
	Any SSc pattern (early/active/late)	4 (7)	9 (13)	30 (17) [14 (14)]	37 (71)	80 (23)
Criteria met post NFC	VEDOSS	5 (9)	0 (0)	36 (21) [16 (16)]	12 (23)	53 (15)
	2013 ACR/EULAR	1 (2)	0 (0)	3 (2) [3 (3)]	37 (71)	41 (12)
	Either SSc criteria	6 (11)	0(0)	39 (23) [19 (19)]	49 (94)	94 (27)

*ACR* American College of Rheumatology, *EULAR* European League Against Rheumatism, *NFC* Nail-fold capillaroscopy, *RP* Raynaud’s Phenomenon, *SSc* Systemic Sclerosis, *VEDOSS* Very early diagnosis of Systemic Sclerosis

Values given indicate *n* (%)

### SSc classification criteria

Following NFC, 49/52 (94 %) patients with SSc and 45/295 (15.3 %) of those without a clinical diagnosis of scleroderma met the VEDOSS or 2013 ACR/EULAR criteria respectively. In particular, 39 (23 %) patients with sRP, including 20 (28 %) patients with CTD or inflammatory arthritis, met either criteria following NFC.

Considering the entire unselected cohort, the detection of a SSc NFC pattern had a sensitivity of 71 % (95 % confidence interval (CI) 61 to 80 %), specificity of 95 % (95 % CI 91 to 97 %), positive predictive value of 84 % (95 % CI 74 to 91 %) and negative predictive value of 90 % (95 % CI 86 to 93 %) for identifying patients who met the VEDOSS or 2013 ACR/EULAR criteria.

## Discussion

To our knowledge this is the first study to demonstrate that the absence of any SSc NFC pattern maintains its known negative predictive value, including in patients with sRP thought to be at increased risk of SSc. As the absence of a SSc NFC pattern is extremely valuable in the exclusion of SSc, NFC could be performed to provide reassurance to the rheumatologist in the assessment of both pRP or sRP.

In line with previous studies, we have demonstrated that the SSc NFC pattern can also be seen in the context of other CTDs. Our study however, and that of Nagy et al [13], are the only two to our knowledge that report NFC findings in an unselected cohort of patients with RP in large numbers; with the remaining studies involving less than 67 patients [11, 12, 14]. As a consequence of these findings, we found some of our patients with non-SSc CTD now met the new SSc classification criteria. It is possible these new criteria may lead to an increase in the prevalence of overlap syndromes.

Interestingly we found that patients meeting the 2013 ACR/EULAR criteria were more likely to have a SSc NFC pattern than those meeting the VEDOSS criteria (84 % vs 42 % respectively). This may be related to the earlier stage of disease in those meeting VEDOSS with less time for detectable vasculopathic changes at the nail fold to develop. These findings are important as the earlier detection and management of SSc may lead to reduced morbidity and earlier detection of its complications.

We acknowledge several limitations with this study. Firstly, we took a pragmatic approach to the NFC examination with no formal measurements taken to determine enlarged/mega capillaries. However, the aim of the study was to capture real-life NFC practice with therefore broader application to the general rheumatologist. Secondly, two different methods of NFC were used, which might have introduced bias. However, as the clinical diagnosis at referral did not influence the method of NFC we feel no selection bias occurred. In addition, different NFC methods are employed by the practising rheumatologists, which we reflect in this report. Also, mirroring standard clinical practice, the NFC examiners were not blinded to the clinical diagnosis, possibly introducing investigator bias. Finally, we acknowledge a retrospective analysis is at risk of missing important data, particularly presence of telangiectasia, however, we would anticipate this study highlights the needs for larger, more defined prospective studies of a heterogeneous group of RP patients.

## Conclusions

To conclude, although a SSc NFC pattern can be found in CTDs other than SSc, we have demonstrated a high specificity for meeting the 2013 ACR/EULAR and VEDOSS criteria for SSc. More importantly, the high negative predictive value of the absence of any SSc NFC pattern in patients with either pRP or sRP is of very high clinical value in the exclusion of SSc.

## Abbreviations

Ab: Antibody
ACA: Anti-Centromere Antibody
ACR: American College of Rheumatology
ANA: Antinuclear Antibody
CTD: Connective Tissue Disease
dcSSc: Diffuse Cutaneous SSc
EULAR: European League Against Rheumatism
IQR: Inter-Quartile Range
lcSSc: Limited Cutaneous SSc
N: Number
NFC: Nail-Fold Capillaroscopy
pRP: Primary Raynaud’s Phenomenon
RP: Raynaud’s Phenomenon
Scl-70: Anti-Topoisomerase Antibody
SD: Standard Deviation
SLE: Systemic Lupus Erythematosus
sRP: Secondary Raynaud’s Phenomenon
SSc: Systemic Sclerosis
UCTD: Undifferentiated Connective Tissue Disease
VEDOSS: Very Early Diagnosis of Systemic Sclerosis
